# Associations between the perceived value of community parks and subjective well-being: a case study in the old town of Nanjing, China

**DOI:** 10.3389/fpubh.2025.1725549

**Published:** 2026-01-09

**Authors:** Guofu Xuan, Jing Zhao, Xinle Wu

**Affiliations:** 1 Department of Tourism Studies, School of Humanities, Southeast University, Nanjing, China; 2 Department of Geography, School of Environment Science, Nanjing Xiaozhuang University, Nanjing, China

**Keywords:** community parks, open green spaces, perceived value, sense of belonging, subjective well-being

## Abstract

**Introduction:**

Open green spaces are crucial for urban sustainability and the psychological well-being of local residents. However, empirical evidence on the psychological benefits of community parks and the underlying mechanisms remains limited. Using community parks in the old town of Nanjing as a case study, this paper examines how the perceived value of community parks influences subjective well-being, as well as the differences between the older and younger groups.

**Methods:**

Data for this study were collected through a carefully designed questionnaire survey aimed at capturing the leisure experiences of community park users. A hypothesized model integrating the perceived value of community parks, sense of belonging, and subjective well-being was constructed and tested. Based on 410 valid questionnaires, structural equation modeling was employed to analyze the model fit, mediating effects, and moderating effects.

**Results:**

The findings identified four dimensions of perceived value and revealed a significant positive correlation between each dimension and subjective well-being, with spatial proximity exerting the strongest effect. Furthermore, the sense of belonging was confirmed to play a mediating role in this relationship. Multi-group analysis indicated that the mechanisms differed between the older and younger groups for three of the nine pathways, while no significant differences were observed for the majority.

**Conclusion:**

This study proposes an explanatory model that elucidates how perceived value impacts subjective well-being, while also highlighting differences between the older and younger groups. These findings emphasize the importance of enhancing perceived value and fostering a sense of belonging, while also meeting the specific needs of older adults to maximize the psychological benefits of community parks. Future research should incorporate additional mediating and moderating variables, and conduct comparative analyses across different regions and cultural contexts.

## 1 Introduction

With the accelerating pace of global urbanization, urban planning and design face the critical challenge of safeguarding residents’ well-being. Urban green spaces are central to addressing this challenge. Open green spaces make fundamental contributions to the socio-ecological functions of modern cities and enhance human well-being through related ecosystem services (1–3). A growing body of cross-sectional and longitudinal epidemiological evidence indicates that exposure to green spaces confers multiple mental health benefits, including stress reduction, attention restoration, depression prevention, alleviation of mental fatigue, and mood enhancement (4–6). Despite the well-documented benefits, consensus on the underlying mechanisms remains elusive (7, 8).

Multiple theoretical pathways have been proposed to explain this positive association. Attention Restoration Theory (ART) and Stress Recovery Theory (SRT) emphasize the direct restorative capacity of green spaces (9, 10). Overall, Attention Restoration Theory emphasizes the cognitive dimension, while Stress Recovery Theory focuses on the affective dimension (9, 10). However, both agree that environments rich in greenery help restore directed attention and promote stress relief.

Another pathway is that green spaces indirectly influence health and well-being through “capacity building” or “harm reduction” (11–13). Urban green spaces provide venues for community leisure and social activities, helping to foster social support and well-being. Green spaces indirectly enhance health and well-being by mitigating the negative impacts of environmental pollution, including air pollution, thermal pollution, and noise pollution (12, 13).

The Ecosystem Services framework is increasingly applied in environmental management and conservation research. This framework defines “the characteristics and functional processes of the natural environment that provide benefits for sustaining and meeting human needs” (4, 14, 15). However, in the field of ecosystem services, the social value of these services and how nature experiences directly impact human mental health have received scant attention (15). Bratman et al. developed a conceptual model integrating mental health into the assessment of ecosystem services based on consensus from natural sciences, social sciences, and health sciences regarding the effects of nature experiences on cognitive function, emotional well-being, and other psychological dimensions (4). The “Natural features-Exposure-Experience-Effects” framework offers a new perspective for research on the relationship between green spaces and mental health.

However, despite a solid theoretical foundation, empirical evidence has yet to reach a consistent conclusion regarding the multiple pathways linking open green space to mental health. First, there are two primary methods for evaluating green spaces: subjective environmental experience and objective environmental exposure (15, 16). Satellite and aerial imaging technologies enable top-down measurement of green space quantities. The most commonly used objective natural exposure indicators include green space area, density, accessibility, and normalized difference vegetative index (NDVI) (17). According to Bratman et al., natural experience is the most central component within the ecosystem services framework, with its quality influenced by natural environmental characteristics and their degree of exposure (4). People’s perceptions and experiences of the natural environment vary from person to person, as reflected in their attitudes toward nature, their level of acceptance, their childhood experiences, and their sense of connection with the natural world (15). Some scholars argue that the most commonly used methods for assessing the relationship between urban public green spaces and human well-being should focus on the perceived experiences of green space users (8). The most commonly used methods for assessing the relationship between urban public green spaces and human well-being primarily focus on studying the perceived experiences of green space users (8). A Chinese study also demonstrated that the objective characteristics of the built environment are more strongly associated with physical and social health, while perceived characteristics have a greater impact on mental health (18).

Second, the theoretical pathways (i.e., mediating variables) have been systematically examined, but the conclusions regarding the path relationships remain inconsistent and even contradictory (4, 19). These inconsistencies may be attributed to differences in study locations and populations, variations in research designs, differing definitions of green space, and variations in green space characteristics (5, 6, 8, 19). Currently, mediating variables receiving greater attention include dimensions such as capacity building (e.g., encouraging physical activity, walking behavior, promoting social cohesion), capacity restoration (e.g., stress reduction), and hazard mitigation (e.g., perceived pollution) (13, 20–22). Some studies suggest that a sense of belonging may be shaped by the environment and potentially impact mental health (23, 24). A sense of belonging reflects the relationship between individuals and their environment, manifesting as a state of integration into a system or setting. It resonates with identity recognition, a feeling of inclusion, and a sense of value (23). Sammie et al. argue that gaining a deep understanding of how different ethnic groups perceive their “sense of belonging” to parks can help park managers accurately identify key areas and target audiences (25).

In summary, although the mental health benefits of green spaces have garnered significant attention, research evidence regarding their underlying relationships and causal pathways remains insufficient, necessitating broader empirical accumulation. Park recreation serves as a vital means of experiencing nature. The interaction between people and parks yields numerous psychological benefits for visitors and the practical implications of this connection are increasingly recognized across various academic disciplines. As a subtype of urban parks, community parks make fundamental contributions to the ecological and social functions of modern cities (23, 26). In recent years, community parks have become increasingly popular in China and worldwide due to their ecological and social roles in urban environments. However, most studies have focused on the benefits and planning of open green spaces in general, with comparatively less attention given to community parks (27, 28). Some researchers have identified the key attributes of community parks, characterizing them as typically small in scale, serving residents from nearby communities, and distinct from large urban parks (29). Community parks have been examined from multiple disciplinary perspectives, including geography, urban planning, psychology, and public health (28). Therefore, examining the role of community parks in promoting local residents’ subjective well-being and elucidating the underlying mechanisms is of considerable importance.

China’s older population has been steadily increasing. By the end of 2023, individuals aged 60 and above accounted for 21.1% of the national population, with the share reaching 24.5% in Jiangsu Province and 21.97% in Nanjing. Due to physical limitations, older adults face greater restrictions on travel distances. Community parks, with their advantages of spatial proximity, ecological services, and opportunities for social interaction, have thus become the primary venues for outdoor recreation among older adults. These parks play a significant role in enriching daily activities and enhancing quality of life for this demographic (30, 31). Older adults also demonstrate distinct characteristics in their leisure motivations, patterns, and experiences (32). Therefore, the mechanisms through which leisure experiences in community parks enhance subjective well-being among older adults, as well as the differences compared to younger adults, warrant further empirical investigation.

Based on the above analysis, we contend that community parks play a vital role in the daily lives of local residents and are crucial for enhancing their well-being, thus warranting specific attention as a distinct type of park. This study takes community parks in the old town of Nanjing as a case study and employs structural equation modeling within the theoretical framework of perceived value–sense of belonging–subjective well-being to investigate the underlying mechanisms through which community parks enhance subjective well-being. By introducing sense of belonging as a mediating variable, the study examines its role in the relationship between perceived value and subjective well-being. In addition, multi-group analysis is conducted to determine whether significant pathway differences exist between the older and younger groups. Through this approach, the study seeks to contribute to the academic literature by establishing an explanatory model of subjective well-being in the context of community parks.

## 2 Literature review and research hypothesis

### 2.1 Perceived value of community parks

As mentioned earlier, the measurement of green space is categorized into objective and subjective measurement methods. Green space attributes closely linked to human perception can significantly impact mental health. Questionnaires are commonly used to measure green space characteristics and assess people’s perceptions of their quantity, quality, accessibility, and interaction patterns. These methods can be complemented by in-depth interviews to comprehensively evaluate the perceived value of green spaces (3, 33, 34).

Enhancing resident well-being is closely linked to the social value of ecosystem services, defined as human perceptions of the quality and benefits derived from natural landscapes (15). As humanity continues to exert increasing pressure on the ecological environment, comprehensive assessments of natural capital and ecosystem services have garnered widespread attention (15, 35). Assessments via field experiments or models may not fully capture social influences, as the social value of ecosystem services is intangible and requires direct experience (35, 36).

The selection of key attributes that directly or indirectly influence perceived value forms the foundation for further analysis. In some representative studies, the assessment of the social value of ecosystem services encompasses 10 aspects: esthetic, Biodiversity, Cultural, Economic, Future, Historical, Life sustaining, Recreation, Spiritual, and Therapeutic (37, 38). Paul categorizes natural experiences into sensory experiences, social experiences, symbolic experiences, and spiritual experiences, with potential overlap among these four types (39). Several studies have contributed to the development of quality assessment models for urban parks, including the Public Open Space Tool (40), the Community Park Audit Tool (41), and the Neighborhood Green Space Tool (29). Some of these models focus on specific types of green spaces (e.g., children’s play areas, neighborhood green spaces) or particular dimensions (e.g., biodiversity, playability). Stessens et al. analyzed the relationship between different features of urban parks and perceived value using an integrated approach, identifying five dimensions as important qualities (quietness, spaciousness, cleanliness and maintenance, facilities, perceived safety) and two as less important (naturalness, historical/cultural value) (42). Similarly, Vasiljević et al. applied factor analysis to extract two perception factors (park condition and park fit-out) from 12 items for peri-urban parks (16).

Based on the analysis of the above studies, this paper summarizes the primary dimensions of park perception evaluation, including environmental quality, emotional value, service facilities, and spatial proximity. It should be noted that spatial proximity is measured objectively in most studies using remote sensing and geographic information system methods. However, research confirms that perceived park distance is positively correlated with multiple exercise outcomes measured objectively (43). Therefore, this study employs subjective assessment to measure spatial proximity.

### 2.2 Sense of belonging to community park

A sense of belonging manifests as a state of integration into a system or environment, encompassing identity recognition, inclusiveness, and a sense of value (44). A sense of belonging may be shaped by the environment and potentially influence mental health, serving as a potential catalyst for parks as cultural ecosystem services. Some scholars believe that social spaces within parks can create more opportunities for users to develop a sense of community identity (45, 46). Research has also found that the mere presence of parks in a community can foster a sense of belonging (47). However, this effect often depends on the users’ perceived value of the park (48, 49). According to Powers et al., both the physical and social environments of a park can influence the development of a sense of belonging (23). Fortune et al. emphasize that creating open and inclusive spaces, providing opportunities for participation, and fostering a welcoming atmosphere are crucial for fostering a sense of belonging (50). Furthermore, differences have been observed between community parks and state parks in their ability to foster a sense of belonging (26). Consequently, Hypothesis 1 is proposed.

*H*1: The perceived value of a community park has a significant positive effect on sense of belonging.

*H*1a: The environmental quality of a community park has a significant positive effect on sense of belonging.

*H*1b: The emotional value provided by a community park has a significant positive effect on sense of belonging.

*H*1c: The service facilities provided by a community park have a significant positive effect on sense of belonging.

*H*1d: The spatial proximity of a community park has a significant positive effect on sense of belonging.

Most scholars recommend using positively worded indicators when measuring the sense of belonging (24, 51). Powers et al. expanded a six-item scale to provide a more comprehensive assessment of welcomeness and belonging (24). In their study, Mullenbach et al. included indicators with statements such as: “I feel welcome in my primary park,” “My primary park is a comfortable place to hang out,” “I feel like I belong at my primary park,” and “At my primary park, I feel I matter” (51). In a comparative study of community and state parks, Powers et al. employed two indicators to evaluate welcomeness and belonging (25). Based on the aforementioned indicator system, the sense of belonging was assessed using five items in this study: “I feel belonged to the park”; “The park is for people like me”; “I feel welcome in this park”; “I feel like I matter at this park”; and “I do not want to move away because of the park.”

### 2.3 Subjective well-being

As a core element of mental health, subjective well-being has received widespread attention due to its significant impact on residents’ quality of life. It is defined as the extent to which individuals evaluate their quality of life positively (6, 30, 52). Since subjective well-being is often influenced by both individual and cultural contexts, it is commonly measured using self-report scales that assess multiple dimensions, including self-rated physical and psychological health, life satisfaction, and eudaimonic well-being, which relates to one’s sense of life purpose (53–55). Self-reported subjective well-being status was measured by happiness, the World Health Organization Well-being Index (WHO-5), and the Short Version of the Warwick-Edinburgh Mental Well-being Scale (SWEMWBS). Among these, the WHO-5 is widely regarded as one of the most sensitive and effective tools for assessing respondents’ subjective well-being and has been validated in previous research (55). This study employed an adapted version of the WHO-5 scale, tailored to the context of community parks and the Chinese population, to measure subjective well-being. A growing body of research has explored the positive outcomes associated with community parks at both the individual and community levels, particularly regarding the physical health and subjective well-being of surrounding urban residents (28). Research on park users’ well-being has grown significantly, with particular attention paid to hedonic well-being, which is associated with life satisfaction (54, 55). As previously discussed, prior research has confirmed that the quality of experiences in parks has a direct or indirect impact on subjective well-being. However, there is no consensus regarding the pathways and mechanisms through which this influence occurs (5, 6, 8, 19). Based on this, this paper proposes the following hypothesis regarding the relationship between experience quality and subjective well-being.

*H*2: The perceived value of a community park has a significant positive effect on subjective well-being.

*H*2a: The environmental quality of a community park has a significant positive effect on subjective well-being.

*H*2b: The emotional value provided by a community park has a significant positive effect on subjective well-being.

*H*2c: The service facilities provided by a community park have a significant positive effect on subjective well-being.

*H*2d: The spatial proximity of a community park has a significant positive effect on subjective well-being.

A sense of belonging, which is viewed as the spiritual characteristic of a place and has been identified as a key aspect of park users’ social relationships, is consequential for subjective well-being (23, 56). Cattell et al. emphasized the importance of social relationships in the well-being benefits derived from residents’ involvement with urban parks (57). According to Van den Bogerd et al., social engagement in proximity to urban open spaces influences residents’ well-being by affecting their sense of belonging (58). Therefore, the sense of belonging to a community park can mediate the effect on subjective well-being.

Based on these insights, we propose Hypothesis 3 and Hypothesis 4:

*H*3: A sense of belonging to a community park has a significant positive effect on subjective well-being.

*H*4: A sense of belonging mediates the relationship between perceived value and subjective well-being.

**Figure 1:**
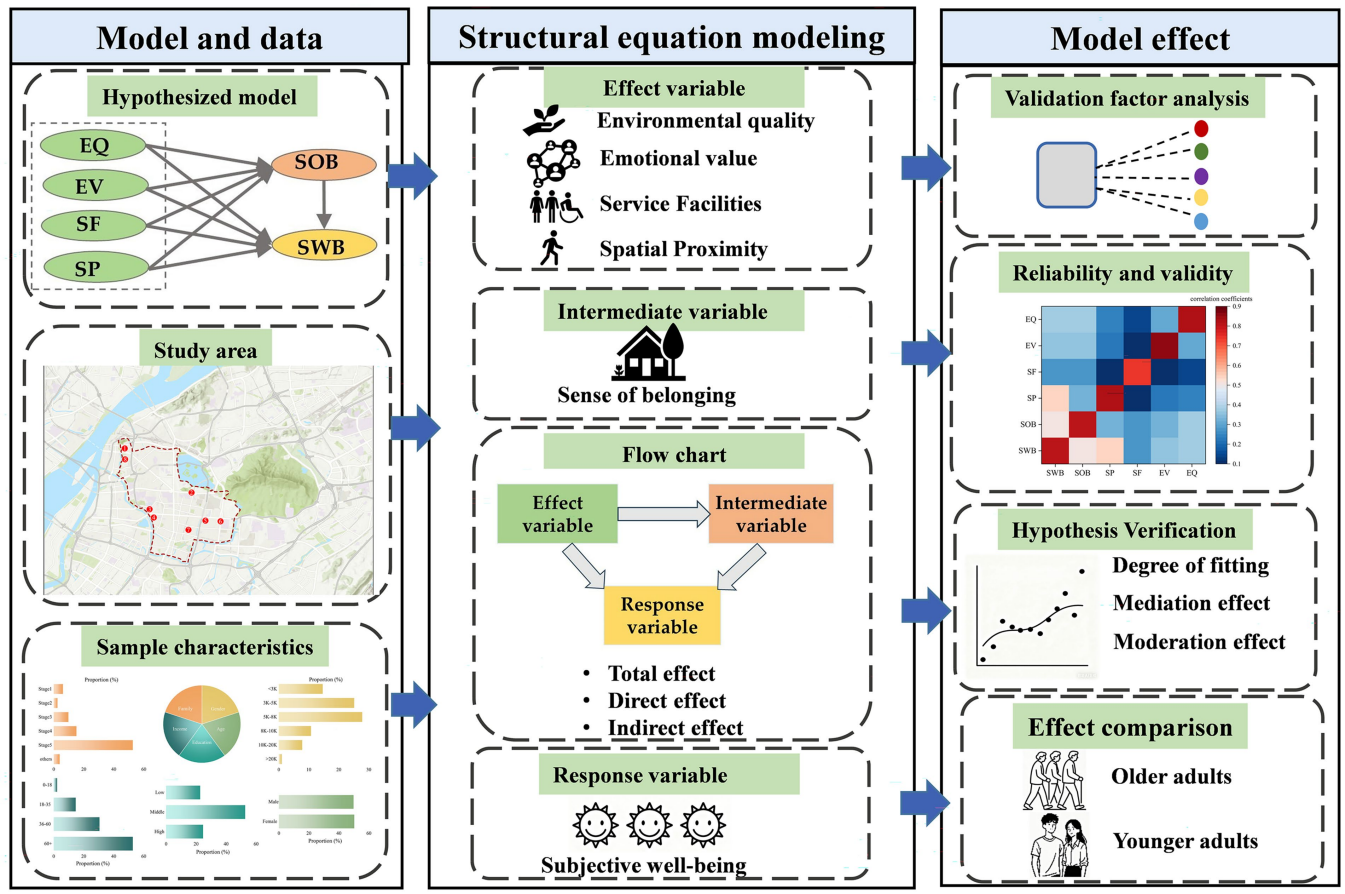
The conceptual framework of the study.

Furthermore, the perceived value of community parks is highly subjective and varies across different demographic groups. Among these groups, the leisure experiences of older adults have received considerable attention in the context of demographic aging (3, 31). Older adults constitute an important user group of community parks, which are small-scale open green spaces designed to serve local residents from neighboring communities (29). Communities with a high proportion of older residents tend to have greater demand for nearby parks. Accordingly, this study examines the differences in pathways and mechanisms between older and younger adults. The research framework of this study is presented in Figure 1.

## 3 Materials and methods

### 3.1 Study area

This study focuses on the old town of Nanjing, the capital of Jiangsu Province and a core city in China’s Yangtze River Delta region. The old town refers to the area enclosed by the Ming Dynasty city walls, with the opposite bank of the moat serving as its boundary. The old town has a rich historical heritage, yet the available land for urban green space development is relatively limited. As a result, community parks characterized by their small scale and proximity to residential areas are particularly important. The “Nanjing Territorial Space Master Plan (2021–2035)” sets a goal of ensuring that a community park is accessible within a 500-meter walk in the old town. Furthermore, the Nanjing Public Facilities Supporting Standards (2023) stipulate that a designated community park should cover 1–2 hectares, with a width of no less than 50 meters and a green space ratio of at least 65%. Moreover, the old town has a high population density and a high proportion of older residents. In light of the prominent mismatch between the supply and demand for green spaces in the old town, the Nanjing municipal government has prioritized the development of small-scale open green areas, such as community and pocket parks.

**Figure 2:**
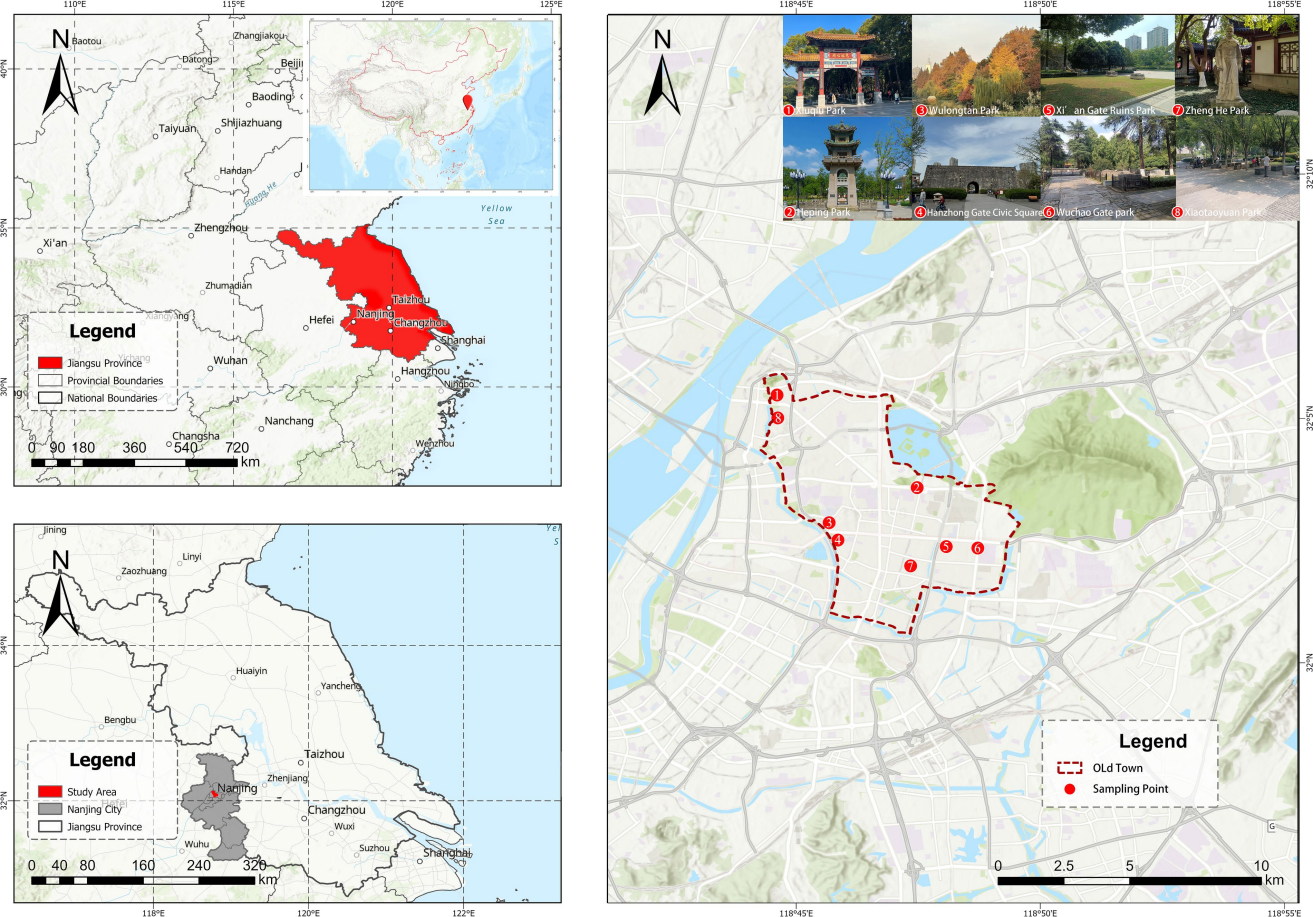
Study area.

Field surveys indicate that, despite the increasing number of small parks constructed within the old town, many suffer from poor service quality and low utilization rates due to inadequate management and delayed maintenance. Consequently, some community parks fail to meet the leisure needs of local residents. Therefore, optimizing the planning and management of such community parks to provide improved leisure services warrants further exploration. According to the Territorial Spatial Master Plan of Nanjing (2021–2035), this study selected eight representative community parks in the old town as sampling points, based on their location and size (see Figure 2).

### 3.2 Questionnaire design

A carefully designed questionnaire survey was conducted to capture the leisure experiences of community park users. The questionnaire, comprising four sections, was developed based on previously validated measurement scales. The first section assessed users’ behavioral characteristics, including transportation modes, travel distance to the park, and visit frequency.

The second section measured leisure involvement and the perceived value of community parks. Perceived value was assessed using 16 items across four dimensions: Environmental Quality (e.g., Unique landscape, Ecological environment, Sanitary conditions, Maintenance), Emotional Value (e.g., Expressing myself, Enhancing relationships with family and friends, Making new friends, Satisfying hobbies), Service Facilities (e.g., Leisure facilities, Rest facilities, Sport facilities, Easy access to toilet, Barrier-free design), and Spatial proximity (e.g., Convenient public transportation, Distance from home, Time required to get here) (42, 59–64). Responses were recorded on a 5-point Likert scale ranging from 1 (strongly disagree) to 5 (strongly agree).

The third section evaluated both the sense of belonging (23, 47, 51, 52) and subjective well-being (2, 55), with each dimension consisting of five items reflecting the psychological benefits derived from community parks. The fourth section collected demographic data, including gender, age, family status, and other relevant characteristics.

### 3.3 Data collection and analysis

To ensure the feasibility of the questionnaire survey, a pre-survey of 50 questionnaires was conducted in November 2024. During the piloting phase, we identified that the phrasing of certain questions was difficult for some respondents, particularly older adults, to comprehend. Additionally, some items were prone to causing confusion. To ensure the validity and clarity of the instrument, these questions were reworded to be more appropriate within the Chinese cultural and linguistic context.

Formal data collection was conducted from February to May 2025, encompassing weekdays, weekends, and public holidays. The schedule was designed to account for variations in visitor patterns, with questionnaires distributed proportionally based on preliminary observations showing that weekend visitation was 2–3 times higher than on weekdays. Surveys were also conducted during the Qingming Festival and Labor Day holidays. Furthermore, daily questionnaire allocation was aligned with visitor flow, focusing on confirmed peak periods (08:00–10:30 and 16:00–18:00). In this study, questionnaires were completed and collected on-site. Prior to distribution, surveyors verified whether respondents were local residents or visitors; only confirmed local residents were given the questionnaires. A total of 450 questionnaires were distributed. After excluding invalid responses (e.g., two answers to the same question, five or more unanswered items across the measurement instruments), 410 valid questionnaires were obtained, resulting in a valid response rate of 91.1%. SPSS 26.0 and AMOS 24.0 were used to analyze the relationships among perceived value, sense of belonging to the community park, and users’ subjective well-being.

## 4 Results

### 4.1 Sample profile

Table 1 summarizes the demographic characteristics of the respondents. The sample exhibited a nearly balanced gender distribution. Individuals aged 60 and above constituted the predominant age group (52.68%), identifying older adults as the primary user demographic. Consistent with this age profile, most respondents held a college degree or lower. The most frequently reported monthly income range was RMB 5,001–8,000 (27.80%), followed by RMB 3,001–5,000 (25.12%), indicating a strong representation of middle-income groups. The largest cohort in terms of family status was married individuals with adult children (52.68%), which aligns with the high proportion of older respondents. In summary, the sample demonstrates diversity in education and income alongside a balanced gender representation, supporting its representativeness and the study’s reliability.

**Table 1:** Basic statistics of respondents.

Attributes	Classification	Frequency (*N* = 410)	Percentage (%)
Gender	Male	204	49.76
	Female	206	50.24
Age	Under 18	9	2.20
	18–35 years old	60	14.63
	36–60 years old	125	30.49
	Over 60 years old	216	52.68
Education	Junior high school and below	93	22.68
	High school/technical school	117	28.54
	Junior college	99	24.15
	College and undergraduate degree	74	18.05
	Postgraduate and above	27	6.59
Monthly income	Under 3000Yuan	60	14.63
	3,001–5000Yuan	103	25.12
	5,001–8000Yuan	114	27.80
	8,001–10000Yuan	44	10.73
	10,000–20000Yuan	32	7.80
	Over 20000Yuan	4	0.98
Family status	Single	25	6.10
	Married without children	10	2.44
	Married with children under the age of 6	40	9.76
	Married with children aged 6 to 18	62	15.12
	Married with children over the age of 18	216	52.68
	others	16	3.90

### 4.2 Measurement model

Reliability testing aims to measure the internal consistency of measurement scales. Typically, a Cronbach’s alpha coefficient exceeding 0.7 indicates good internal consistency and high reliability. The results showed that the Cronbach’s alpha coefficients for environmental quality, emotional value, service facilities, spatial proximity, sense of belonging, and subjective well-being were 0.896, 0.916, 0.861, 0.856, 0.907, and 0.904, respectively (Table 2), indicating strong reliability of the measurement model.

**Table 2:** Confirmatory factor analysis results.

Codes	Dimensions and items	Factor loadings	CR	AVE	Cronbach’s α
	Environmental quality		0.897	0.687	0.896
EQ1	Unique landscape	0.865			
EQ2	Ecological environment	0.849			
EQ3	Sanitary conditions	0.854			
EQ4	Maintenance	0.740			
	Emotional value		0.916	0.732	0.916
EV1	Expressing myself	0.891			
EV2	Enhancing relationships with family and friends	0.821			
EV3	Making new friends	0.854			
EV4	Satisfying hobbies	0.854			
	Service facilities		0.862	0.555	0.861
SF1	Leisure facilities	0.764			
SF2	Rest facilities	0.768			
SF3	Sport facilities	0.730			
SF4	Easy access to toilet	0.742			
SF5	Barrier-free design	0.719			
	Spatial proximity		0.858	0.669	0.856
SP1	Convenient public transportation	0.880			
SP2	Distance from home	0.827			
SP3	Time required to get here	0.741			
	Sense of belonging		0.907	0.662	0.907
SOB1	I feel belonged to the park	0.892			
SOB2	The park is for people like me	0.819			
SOB3	I feel welcome in this park	0.852			
SOB4	I feel like I matter at this park	0.751			
SOB5	I do not want to move away because of the park	0.745			
	Subjective well-being		0.905	0.656	0.904
SWB1	I feel comfortable and pleasant in this park	0.864			
SWB2	I feel stress recovery in this park	0.867			
SWB3	Enjoyed my time in this park enrich my life	0.770			
SWB4	Recreation in this park improve my health	0.725			
SWB5	Recreation in this park make me feel satisfied with life	0.815			

Prior to hypothesis testing, confirmatory factor analysis (CFA) was conducted to assess the construct validity and reliability of the measurement model. The model demonstrated a good fit: χ^2^/df = 2.566, RMSEA = 0.062, CFI = 0.935, GFI = 0.862, TLI = 0.926, IFI = 0.936, NFI = 0.899, and SRMR = 0.050. These indices confirm that the overall fit of the measurement model meets acceptable standards. The statistics presented in Table 2 indicate that the factor loadings ranged from 0.719 to 0.892, all above 0.7, meeting the recommended thresholds (65). The composite reliability (CR) for each construct surpassed 0.7, and the average variance extracted (AVE) for all latent variables exceeded 0.5, thereby meeting the established standards for convergent validity (65). In this study, the CR values for environmental quality, emotional value, service facilities, spatial proximity, sense of belonging, and subjective well-being were 0.897, 0.916, 0.862, 0.858, 0.907, and 0.905, respectively. The corresponding AVE values were 0.687, 0.732, 0.555, 0.669, 0.662, and 0.656.

Discriminant validity was assessed by comparing the square root of the average variance extracted (AVE) with the correlation coefficients for each latent construct. If the square root of the AVE exceeds the correlation coefficients, it indicates satisfactory discriminant validity between the latent variables. The second column on the left side of Table 3 presents the AVE values for each latent variable. The bolded values represent the square roots of the AVE, while the remaining values correspond to the correlation coefficients between the latent variables. As shown in Table 3, the correlation coefficients among the latent variables ranged from 0.101 to 0.539, all of which are lower than the square roots of the AVE. This indicates that the latent variables are sufficiently distinct and that the questionnaire possesses satisfactory discriminant validity.

**Table 3:** Discriminant validity assessment and correlation matrix.

Constructs	AVE	SWB	SOB	SP	SF	EV	EQ
SWB	0.656	**0.810**					
SOB	0.662	0.514	**0.814**				
SP	0.669	0.539	0.332	**0.818**			
SF	0.555	0.282	0.270	0.101	**0.745**		
EV	0.732	0.365	0.332	0.233	0.110	**0.856**	
EQ	0.687	0.382	0.372	0.239	0.138	0.301	**0.829**

EQ, Environmental Quality; EV, Emotional Value; SF, Service Facilities; SP, Spatial Proximity; SOB, Sense of Belonging; SWB, Subjective Well-being. The bold values indicate the square roots of the AVE for each construct.

### 4.3 Structural modeling and hypothesis verification

**Figure 3:**
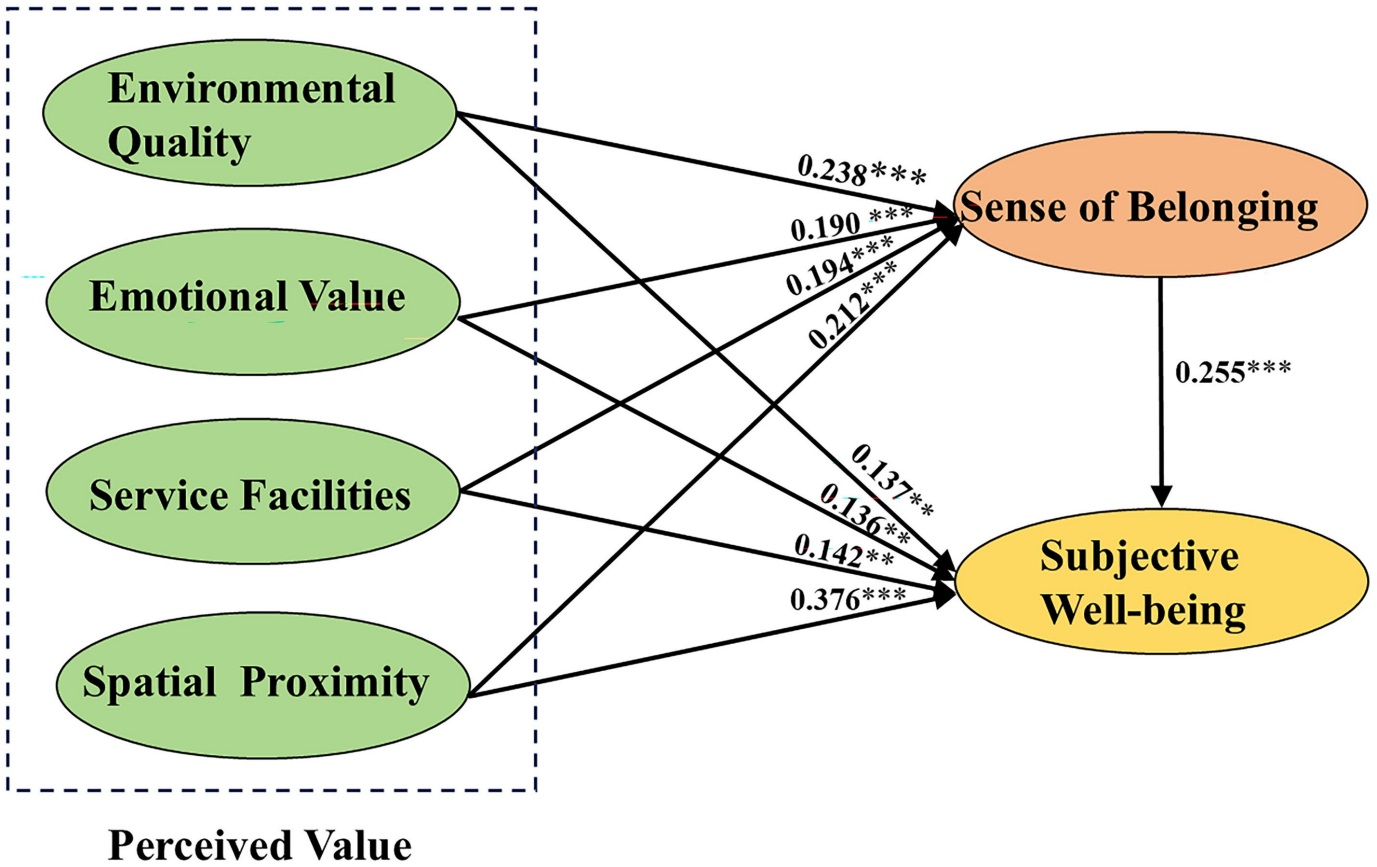
Results of the overall structural equation modeling (***p < 0.001; **p < 0.01; *p < 0.05).

The SEM was applied to validate the hypothesized model and results of the structural model are presented in Figure 3. The analysis revealed significant positive relationships between the dimensions of perceived value and users’ sense of belonging. Specifically, environmental quality (*β* = 0.238, *p* < 0.001), emotional value (*β* = 0.190, *p* < 0.001), service facilities (*β* = 0.194, *p* < 0.001), and spatial proximity (*β* = 0.212, *p* < 0.001) all significantly and positively influenced sense of belonging, thus supporting H1a, H1b, H1c and H1d. A comparison of the path coefficients indicated that environmental quality had the strongest effect on sense of belonging, followed by spatial proximity.

The standardized path coefficients for H2a (*β* = 0.137, *p* < 0.01), H2b (*β* = 0.136, *p* < 0.01), H2c (*β* = 0.142, *p* < 0.01), and H2d (*β* = 0.376, *p* < 0.001) indicating hypotheses H2a, H2b, H2c, and H2d are verified. A significant positive relationship between the perceived value of community parks and subjective well-being. This demonstrates that environmental quality, emotional value, service facilities, and spatial proximity all pronouncedly affect community park users’ subjective well-being.

Regarding H3, the coefficient is 0.255 (*p* < 0.001), demonstrating that sense of belonging also serves as a significant predictor of community park users’ subjective well-being. Thus, hypothesis H3 is supported.

### 4.4 Mediation role of sense of belonging

To examine the mediating role of sense of belonging, a bootstrapping analysis was conducted with 5,000 iterations at a 95% confidence level. Both percentile and bias-corrected confidence intervals (CIs) were examined; the mediation effect was considered significant if the intervals excluded zero. The results are presented in Table 4.

**Table 4:** Results of mediation analysis.

Hypothesis paths	Path effects	Size of effect	S.E.	t-value	Bias-corrected 95% CI			Percentile 95% CI		
					Lower	Upper	*p*	Lower	Upper	*p*
EQ → SWB	Total	0.225	0.071	3.169	0.088	0.368	**	0.082	0.362	**
	Direct	0.156	0.072	2.167	0.018	0.300	*	0.014	0.296	*
	Indirect	0.069	0.030	2.300	0.023	0.140	***	0.020	0.136	**
EV → SWB	Total	0.194	0.063	3.079	0.068	0.315	**	0.070	0.318	**
	Direct	0.143	0.062	2.306	0.017	0.261	*	0.021	0.266	*
	Indirect	0.051	0.024	2.125	0.014	0.111	**	0.012	0.106	**
SF → SWB	Total	0.275	0.082	3.354	0.123	0.451	***	0.127	0.457	***
	Direct	0.204	0.084	2.429	0.045	0.376	*	0.049	0.380	*
	Indirect	0.071	0.031	2.290	0.022	0.147	**	0.020	0.142	**
SP → SWB	Total	0.469	0.066	7.106	0.342	0.605	***	0.340	0.602	***
	Direct	0.410	0.073	5.616	0.269	0.554	***	0.268	0.552	***
	Indirect	0.059	0.028	2.107	0.016	0.129	*	0.014	0.123	*

EQ, Environmental Quality; EV, Emotional Value; SF, Service Facilities; SP, Spatial Proximity; SOB, Sense of Belonging; SWB, Subjective Well-being. ***p < 0.001; **p < 0.01; *p < 0.05.

For the path from environmental quality to subjective well-being, the indirect effect was significant (*β* = 0.069), as both the bias-corrected (0.023–0.140) and percentile (0.020–0.136) 95% CIs excluded zero. The direct effect was also significant (bias-corrected CI: 0.018–0.300; percentile CI: 0.014–0.296).

Regarding emotional value, the indirect effect on subjective well-being was significant (*β* = 0.051; bias-corrected CI: 0.014–0.111; percentile CI: 0.012–0.106). A significant direct effect was also present (bias-corrected CI: 0.017–0.261; percentile CI: 0.021–0.266).

For service facilities, both the direct (bias-corrected CI: 0.045–0.376; percentile CI: 0.049–0.380) and indirect effects (*β* = 0.071; bias-corrected CI: 0.022–0.147; percentile CI: 0.020–0.142) were significant.

Concerning spatial proximity, the indirect effect was significant (*β* = 0.059; bias-corrected CI: 0.016–0.129; percentile CI: 0.014–0.123). The direct effect was also significant (bias-corrected CI: 0.269–0.554; percentile CI: 0.268–0.552).

Since all four dimensions of perceived value exhibited significant direct and indirect effects, sense of belonging partially mediates the relationship between perceived value and subjective well-being. Therefore, Hypothesis H4 is supported.

### 4.5 Multi-group analysis

**Figure 4:**
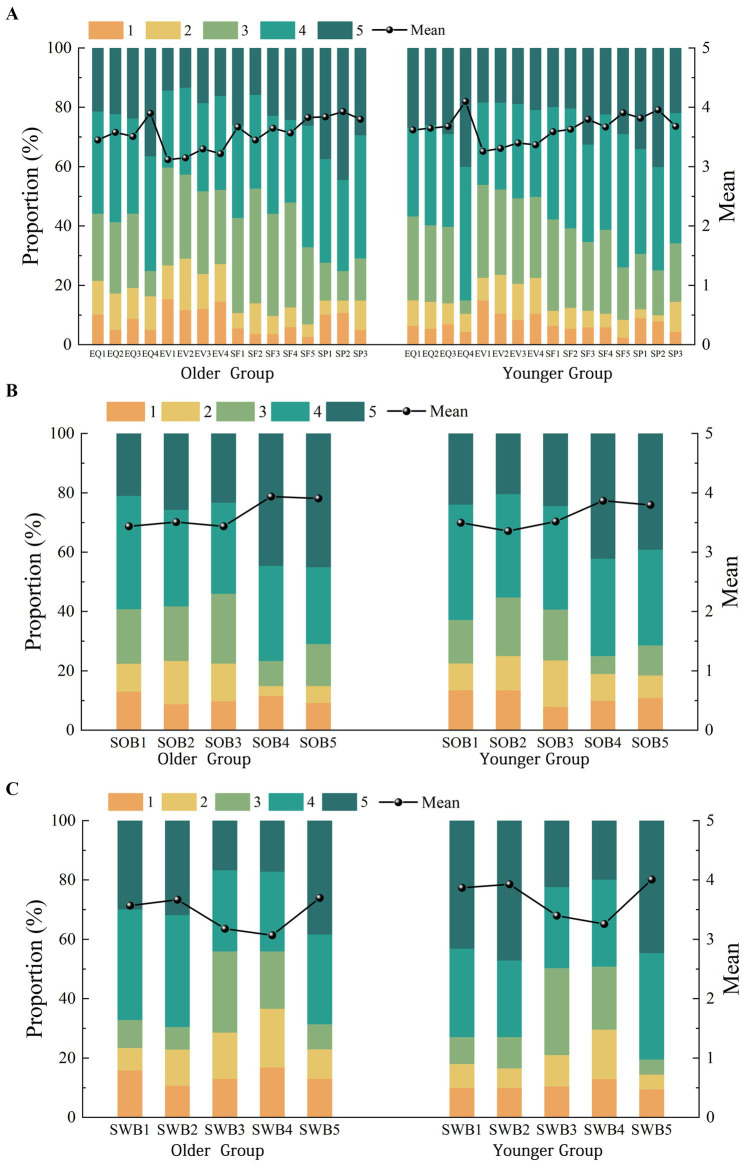
Distributions of perceived value **(A)**, sense of belonging **(B)**, and subjective well-being **(C)** by age group.

Descriptive statistics for the older and younger samples are presented in Figure 4. For both the older and younger groups, evaluation scores across most dimensions of perceived value predominantly ranged from 3 to 4. The mean values for the older group were slightly lower than those for the younger group on most indicators of perceived value, except for SF1 (Leisure facilities), SP1 (Convenient public transportation), and SP3 (Time required to get here). In terms of sense of belonging, the older group scored higher on SOB2 (The park is for people like me), SOB4 (I feel like I matter at this park), and SOB5 (I do not want to move away because of the park), whereas the younger group scored slightly higher on SOB1 (I feel belonged to the park) and SOB3 (I feel welcome in this park). Significant differences were observed between the two groups in subjective well-being, with the younger group scoring higher across all indicators.

To examine the moderating effect of age, respondents were categorized into two groups: the older group (over 60 years old, *N* = 212) and the younger group (under 60 years old, *N* = 198). First, an invariance test was conducted to assess the stability of the proposed model across the age groups. The unconstrained and constrained models were compared using AMOS multi-group analysis. As presented in Table 5, the goodness-of-fit indices for both unconstrained model (χ^2^/df = 1.973, RMSEA = 0.049, CFI = 0.921, TLI = 0.910, IFI = 0.922, GFI = 0.814) and constrained model (χ^2^/df = 1.952, RMSEA = 0.048, CFI = 0.920, TLI = 0.912, IFI = 0.921, GFI = 0.810) were acceptable, indicating that the model adequately fit the data and could be used to test the hypothesized relationships among the variables.

**Table 5:** Model fit indices for measurement invariance tests.

Model	χ^2^	df	χ^2^/df	RMSEA	CFI	TLI	IFI	GFI	△χ^2^	Invariant measurement
Unconstrained model	1120.556	568	1.973	0.049	0.921	0.910	0.922	0.814	△χ^2^ (20) = 27.335, *p* = 0.126>0.05	Supported
Constrained model	1147.891	588	1.952	0.048	0.920	0.912	0.921	0.810		

To examine whether significant differences existed in the hypothesized paths between the age groups, the estimated parameter coefficients were constrained to be equal across groups, and the chi-square difference between the models was examined. The results indicated no significant difference between the models (△χ^2^(20) = 27.335, *p* = 0.126 > 0.05), suggesting that age grouping did not affect the applicability of the model. Therefore, subsequent path analyses could be conducted using the hypothesized model across both age groups.

**Figure 5:**
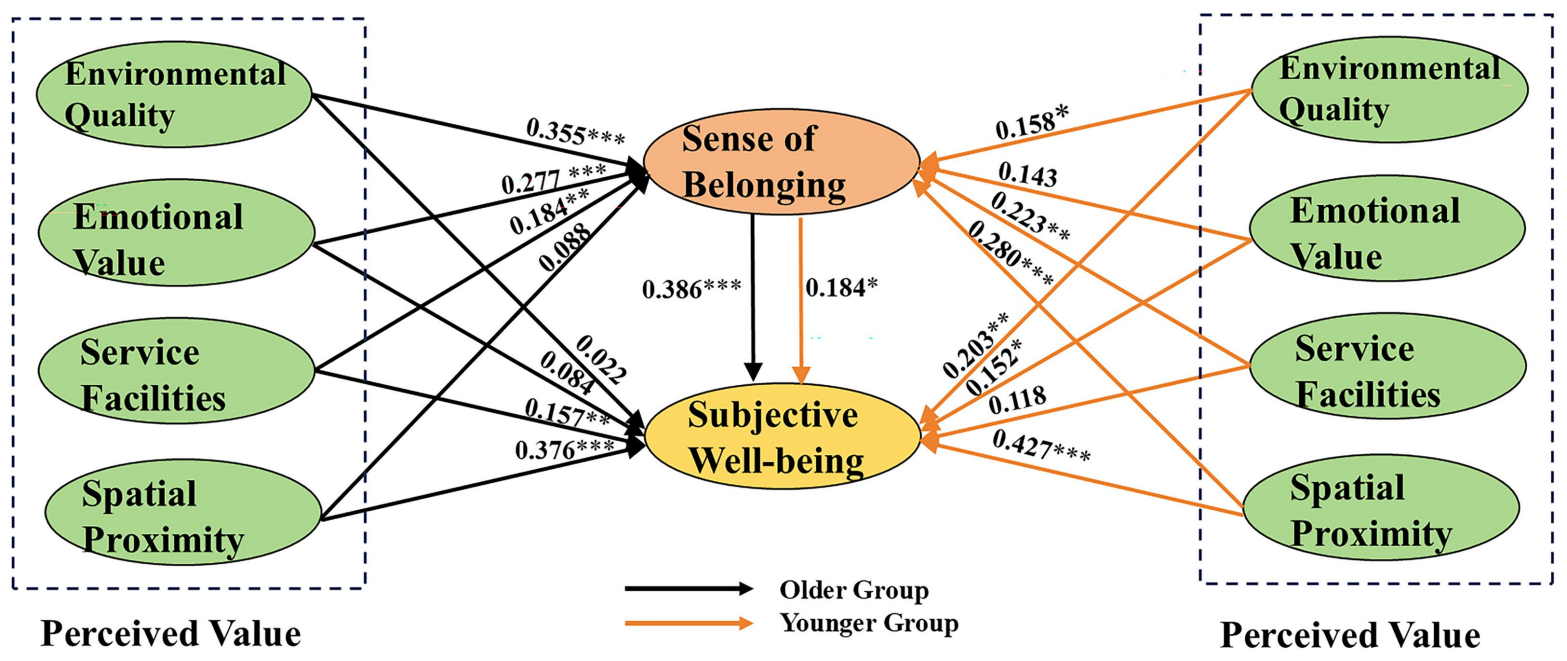
Comparison of path coefficients between age groups.

A multi-group SEM analysis was conducted to examine the moderating effect of age. Results from the synchronous model revealed a significant difference between the older and younger groups: Δχ^2^ = 76.833, Δdf = 55, *p* = 0.028 (< 0.05). The differences in each path are presented in Table 6 and Figure 5, with the older and younger groups exhibiting significant differences in three of the nine paths of the hypothesized model: Spatial Proximity→ Sense of Belonging, Environmental Quality →Subjective Well-Being, and Sense of Belonging →Subjective Well-Being. The path coefficient for the influence of spatial proximity on sense of belonging was higher in the younger group (0.280) than in the older group (0.088). Similarly, the path coefficient for the effect of environmental quality on subjective well-being was greater in the younger group (0.203) compared to the older group (0.022). Among the total effects, differences were also observed in the path from sense of belonging to subjective well-being, with the coefficient being higher for the older group (0.386) than for the younger group (0.184). However, age did not significantly moderate the other path relationships.

**Table 6:** Results of multi-group structural equation modeling.

Hypothesis paths	Path coefficients (Older)	T-value	Path coefficients (Younger adults)	T-value	Critical ratio of coefficient difference	Significant difference
EQ→SOB	0.355***	4.88	0.158*	2.06	−1.256	Unsupported
EV→SOB	0.277***	3.92	0.143	1.91	−1.076	Unsupported
SF→SOB	0.184**	2.75	0.223 **	2.94	0.49	Unsupported
SP→SOB	0.088	1.19	0.280 ***	3.74	2.092	Supported
EQ→SWB	0.022	0.34	0.203**	2.86	2.038	Supported
EV→SWB	0.084	1.31	0.152*	2.23	0.77	Unsupported
SF→SWB	0.1657**	2.60	0.118	1.71	−0.447	Unsupported
SP→SWB	0.376***	5.37	0.427***	5.62	0.822	Unsupported
SOB→SWB	0.386***	5.14	0.184 *	2.53	−2.126	Supported

EQ, Environmental quality; EV, Emotional Value; SF, Service Facilities; SP, Spatial Proximity; SOB, Sense of Belonging; SWB, Subjective Well-being. ***p < 0.001; **p < 0.01; *p < 0.05.

**Figure 6:**
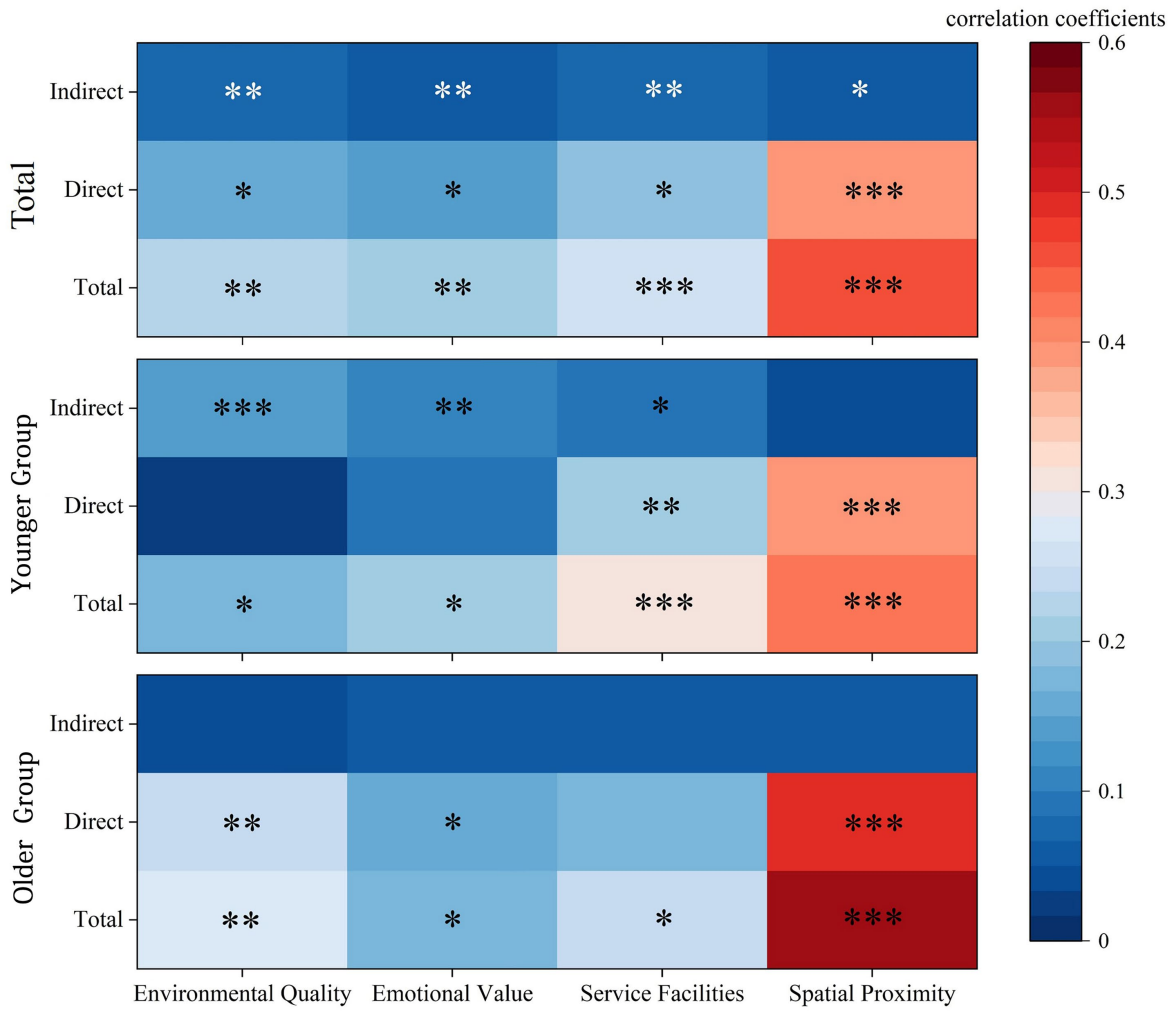
Heat map of correlation coefficients.

The comparative analysis of direct, indirect, and total effects in Figure 6 further highlights the distinct age differences in the mechanisms linking perceived value to subjective well-being in community parks. Compared with the younger group, the indirect effects were more pronounced among the older group, indicating that fostering a sense of belonging is particularly crucial for enhancing subjective well-being during leisure activities in community parks for older adults. For the older group, the direct effects that played a significant role were those of service facilities and spatial proximity.

## 5 Discussion

### 5.1 Theoretical implications

This study deepens the understanding of the relationship between perceptions of urban green spaces and mental health. Consistent with previous cross-sectional research, our findings confirm a significant positive correlation between the perceived value of community parks and subjective well-being (45, 47, 53, 66). The perceived value of community park experiences significantly enhances residents’ subjective well-being, further validating classic theories such as Attention Restoration Theory (ART), Stress Reduction Theory (SRT), and Ecosystem Services Theory.

This study employs the perceived value of community parks as a subjective assessment metric for green spaces. The good model fit confirms that the subjective evaluation of green spaces is a significant determinant of subjective well-being. This further demonstrates that incorporating “experience” as a core component within ecosystem service models is highly justified (4, 35, 36, 38).

The perceived value assessed in this study encompasses four dimensions: environmental quality, emotional value, service facilities, and spatial proximity. Among the four dimensions of perceived value, spatial proximity is the strongest predictor. The notable influence of spatial proximity suggests that easy access to community parks significantly enhances users’ subjective well-being, which is consistent with findings from other studies (2, 52, 53). Issues of spatial accessibility and environmental justice are currently of significant concern and sensitivity to residents in China. Among the other three dimensions, environmental quality and service facilities are also frequently addressed in other studies, consistently indicating a positive association with subjective well-being (54, 55, 57). Emotional value, previously underrepresented in green space assessments, was incorporated into this study’s perceived value evaluation. This inclusion draws upon research on the social value of ecosystem service systems (15, 35, 38). It exhibits a significant positive correlation, indicating that the experience of green spaces constitutes a comprehensive and multifaceted process.

A sense of belonging reflects an individual’s emotional attachment to a place and is a crucial manifestation of the relationship between people and their environment. By identifying sense of belonging as a mediator, this study provides new insights by delineating the specific pathway through which perceived value enhances subjective well-being. The results indicate that a sense of belonging to community parks partially mediates the relationship between perceived value and subjective well-being. As some scholars have pointed out, urban natural environments may play a role in fostering a sense of belonging and community attachment (67, 68). These emotional experiences, in turn, can nurture “key elements” that influence psychological well-being, such as stability, familiarity, and security (69). However, the relationship between natural exposure, place attachment, and well-being remains understudied (70). Therefore, it is necessary to strengthen this perspective in future research.

Furthermore, this study uses multi-group analysis to examine pathway differences between the older and younger groups. In terms of direct effects, the dimensions of perceived value with significant influence for older adults are service facilities and spatial proximity, suggesting that older adults place greater importance on the adequacy of park facilities and the proximity of parks to their residences. As people age, their mobility typically declines and their range of activities becomes more restricted. At the same time, their social networks tend to shrink due to changes in social circumstances and health conditions. Therefore, green spaces within their immediate living environment are crucial for meeting their needs (3, 29, 31).

The findings indicate that the effect of sense of belonging on well-being is more pronounced for older adults. The indirect effects were also significantly stronger for older adults. Specifically, for older adults, a sense of belonging developed through leisure activities and social interactions in community parks is critical for enhancing subjective well-being. Other case studies have also found that older adults exhibit greater familiarity and place attachment toward surrounding green spaces (71). High-quality green spaces not only enhance social interaction among neighbors but also foster stronger community bonds for older adults (49). Community parks create opportunities for social engagement, strengthen social connections, and enhance a sense of belonging. The benefits of social interaction for older adults are well-documented in research, showing positive correlations with improved health status, well-being, and quality of life (72).

However, no significant differences were observed between the older and younger groups in six of the nine pathways, suggesting limited age-group differences in the mechanisms linking perceived value to subjective well-being in community parks. These findings partially align with those of Tinsley et al., who argued that age differences are less influential than racial and social environmental factors in shaping well-being (32). Future research should further investigate these distinctions to reconcile potential variability in findings across different methodological approaches and cultural contexts.

### 5.2 Managerial implications

Given the growing focus on addressing the rising prevalence of mental health issues through urban green space interventions and enhancing the well-being of expanding urban populations, the findings of this study hold significant practical implications.

It is essential for urban planners and policymakers to manage community parks with a user-centric approach. This study confirms that perceived value significantly enhances users’ subjective well-being, offering practical recommendations for enhancing the usability of community parks and promoting social equity. The perceived value of community parks is shaped by factors such as environmental quality, emotional value, service facilities, and spatial proximity. These factors not only address users’ leisure needs but also foster social interaction, ultimately improving users’ subjective well-being.

Among the dimensions of perceived value, spatial proximity has the most pronounced impact on subjective well-being. Therefore, prioritizing the development of small-scale urban parks—such as community parks and pocket parks—is crucial, particularly in the old town. Establishing a community park system with scientifically planned accessibility and equitable spatial distribution is critical for achieving both psychological benefits and social equity. Environmental quality is another key factor enhancing subjective well-being in community parks. By implementing strategies that promote attractive landscapes and inclusive environments, urban planners can substantially increase park usage and maximize psychological benefits for users.

A sense of belonging to community parks plays a critical mediating role in shaping users’ subjective well-being. By expanding and diversifying specific amenities (e.g., versatile activity lawns, well-maintained walking paths) and leisure activities (e.g., traditional festival theme activities, opera corners), community parks can increase user participation, thereby strengthening their sense of belonging. Consequently, park design that fosters social interaction and encourages leisure participation can enhance this sense of belonging, ultimately boosting the subjective well-being of surrounding residents. This study revealed that older adults reported relatively lower perceived value and subjective well-being compared to younger adults. In the context of demographic aging, the leisure needs of older adults warrant greater attention. Our research confirms that among the various pathways influencing subjective well-being, the direct and indirect effects of sense of belonging are significantly stronger for older adults than for younger adults. This underscores that fostering a sense of belonging through park-based recreation and social interaction serves as a key mechanism for enhancing subjective well-being in later life. Given the significant mediating role of sense of belonging in the model, integrating age-friendly facilities and promoting social space design (e.g., sheltered seating areas and community gardens) in community park planning to actively support the improvement of older adults’ subjective well-being emerges as an important practical implication.

### 5.3 Limitations and future research

This study has several limitations that should be acknowledged and addressed in future research. First, due to the unique cultural context of China and the specific characteristics of the old town of Nanjing, this study examined a limited set of factors influencing community park users’ subjective well-being. Other variables that may significantly affect subjective well-being were not included. Future research should incorporate additional factors to enrich the explanatory model of subjective well-being in community parks. Although this study confirms that a sense of belonging mediates the relationship between perceived value and subjective well-being, other potential mediating variables may exist and warrant further investigation. Additionally, although a moderating effect of age was identified, the observed differences between the older and younger groups were relatively limited. Future research should broaden the scope to examine other potential moderating variables, such as gender, education, income, and family status.

Second, data collection in this study was limited to several community parks in the old town, which restricts the generalizability of the research findings. Previous studies indicate that findings can vary across regions with different cultures. Therefore, future research should broaden the geographical and cultural scope to facilitate comparative analyses. Additionally, future research should examine whether the proposed framework is specific to community parks or can be generalized to other park types. This will contribute to establishing a specialized research framework tailored for community parks.

Third, this study relied on subjective assessments to measure perceived value, and while their validity was demonstrated, the lack of objective quantification of green spaces remains a limitation. Future research should integrate both objective and subjective metrics to capture their complementary insights. Additionally, as subjective well-being is multidimensional, findings may vary with different measurement scales, underscoring the need for further in-depth research and empirical accumulation.

## 6 Conclusion

By constructing a structural equation model incorporating mediating effects, this study examined the direct and indirect influences of perceived value on subjective well-being within community parks, with particular attention to the differences between the older and younger groups. Based on empirical research conducted in Nanjing, the key findings are summarized as follows:

A positive correlation exists between the perceived value of community parks and users’ subjective well-being. Environmental quality, emotional value, service facilities, and spatial proximity all significantly influence users’ subjective well-being. Among these dimensions, spatial proximity exerts the strongest positive effect. Therefore, enhancing the accessibility of community parks is crucial for improving users’ subjective well-being.

This study also confirmed the mediating role of a sense of belonging in the relationship between perceived value and subjective well-being. Across all dimensions of perceived value, the mediating effect of sense of belonging was significant. Accordingly, the study delineates a pathway from perceived value to users’ subjective well-being through sense of belonging. For policymakers, it is essential to prioritize park attributes that promote social interaction and strengthen the sense of belonging.

Furthermore, multi-group analysis revealed significant differences between the older and younger groups on three of the nine pathways. The effect of sense of belonging on subjective well-being was more pronounced for the older group. However, no significant differences were observed between the older and younger groups in six out of nine pathways, suggesting that the mechanisms influencing subjective well-being in community parks are largely similar across these age groups.

## Data Availability

The original contributions presented in the study are included in the article/supplementary material, further inquiries can be directed to the corresponding author.
